# Risk factors for early septic failure after two-stage exchange total knee arthroplasty for treatment of periprosthetic joint infection

**DOI:** 10.1186/s10195-024-00750-w

**Published:** 2024-02-12

**Authors:** Woo-Suk Lee, Kwan Kyu Park, Byung-Woo Cho, Jun Young Park, Inuk Kim, Hyuck Min Kwon

**Affiliations:** 1 Department of Orthopaedic Surgery, Severance Hospital, Yonsei University College of Medicine, Seoul, Republic of Korea; 2 Department of Orthopaedic Surgery, Gangnam Severance Hospital, Yonsei University College of Medicine, Seoul, Republic of Korea; 3 Department of Orthopaedic Surgery, Yongin Severance Hospital, Yonsei University College of Medicine, Gyeonggi-do, Republic of Korea

**Keywords:** Two-stage exchange total knee arthroplasty, Septic failure, Periprosthetic joint infection, Reimplantation

## Abstract

**Background:**

The cause of early septic failure after two-stage exchange revision total knee arthroplasty (TKA) for chronic periprosthetic joint infection (PJI) and the factors affecting it are not well known. The purpose of this study was to determine the surgical outcomes and the risk factors for early septic failure after two-stage revision TKA for chronic PJI.

**Methods:**

We identified a total of 246 adult patients who met the Musculoskeletal Infection Society (MSIS) diagnostic criteria for chronic PJI at two academic tertiary hospitals from March 2012 to December 2018. Finally, 151 patients who consecutively received two-stage exchange revision TKA for chronic PJI and who had a minimum 3-year follow-up were enrolled and retrospectively reviewed. Successful surgical treatment was evaluated for two-stage revision TKA and risk factors for early septic failure were identified.

**Results:**

Early septic failures occurred within 3 years after reimplantation in 48 patients (31.8%). After accounting for potentially confounding variables, we found that male patient [odds ratio (OR): 2.753, 95% confidence interval (CI) 1.099–6.893, *p* = 0.031], fungus or mycobacterial infection (OR: 5.224, 95% CI 1.481–18.433, *p* = 0.01), and positive culture at reimplantation (OR: 4.407, 95% CI 1.255–15.480, *p* = 0.021) were independently associated with early septic failure after two-stage exchange revision TKA.

**Conclusion:**

Male patients, fungus or mycobacterial infection, and positive culture at reimplantation were independently associated with an increased risk of early septic failure after two-stage exchange revision TKA despite normal C-reactive protein values prior to reimplantation. Further prospective and high-quality studies are needed to determine the risk factors of two-stage exchange revision TKA for chronic PJI.

*Level of evidence*: level IV; retrospective comparison; treatment study.

## Introduction

Periprosthetic joint infection (PJI) following total knee arthroplasty is a serious complication that is accompanied by high morbidity and mortality  [1–3]. Recently, Lum et al. reported a mean mortality rate of 14.4% with an average follow-up of 3.8 years in a meta-analysis. [4]. Several surgical treatment options are available for PJI such as debridement, antibiotics and implant retention (DAIR), one-stage exchange revision, two-stage exchange revision, and salvage procedures (arthrodesis or amputation) [5, 6]. Among these, two-stage exchange revision total knee arthroplasty is currently the gold standard treatment for chronic PJI after total knee arthroplasty. [2, 7–11].

Because early septic failure after two-stage exchange of PJI is associated with higher complication rates and comorbidities, successful eradication of PJI after total knee arthroplasty is very important [12–14]. Although several studies have reported good infection-free survival rates after two-stage exchange at mid-term follow-up [15, 16], and reimplantation is performed when it is determined that the infection is completely eradicated with serologic normal C-reactive protein (CRP) values, the reinfection rate is still high, ranging from 10% to 40% [13, 17–19]. In the case of early septic failure after two-stage exchange revision, poor prognosis and infection recurrence would be expected after additional surgical treatment; therefore, socioeconomic burden is considerable [20–22].

There may be various underlying factors for early septic failure, including surgical factors, microbiology, antibiotics, as well as patient factors such as immune activity, which affect sepsis in a complex way  [9, 23]. However, to the best of our knowledge, the cause of early septic failure after two-stage exchange revision TKA for chronic PJI, and the factors affecting failure, are not well known. Therefore, we aimed to (1) investigate the surgical outcomes of two-stage exchange revision TKA for chronic PJI and (2) analyze the risk factors for early septic failure after two-stage exchange revision TKA for chronic PJI.

## Methods

### Patient selection

**Fig. 1:**
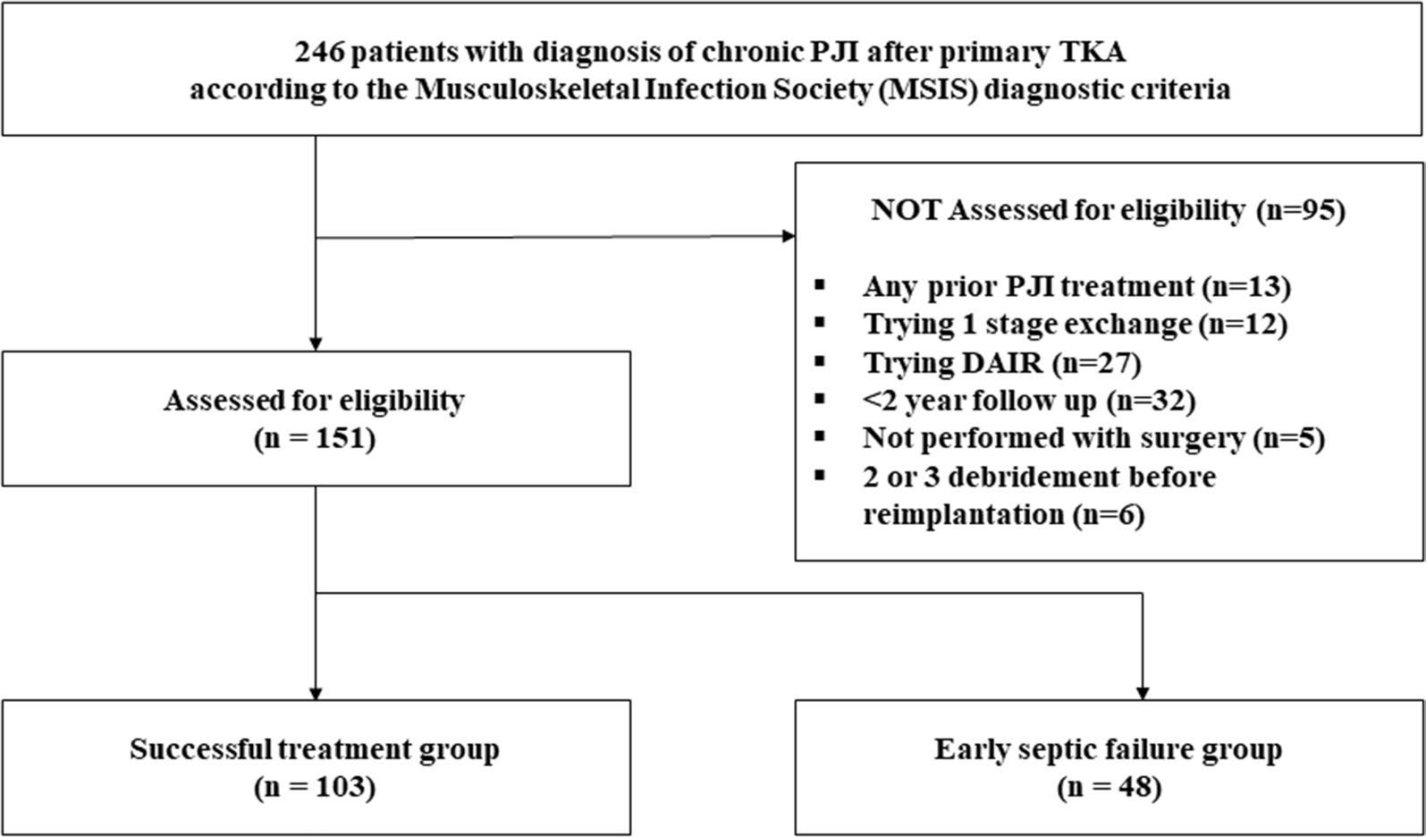
Flowchart of patient inclusion

After institutional review board (IRB) approval was obtained, from March 2012 to December 2018, a total of 246 adult patients who met the Musculoskeletal Infection Society (MSIS) diagnostic criteria for chronic PJI in two academic tertiary hospitals were identified [24]. Patients who had any prior PJI treatment history (*n* = 13), who underwent one-stage exchange total knee arthroplasty (*n* = 12) or DAIR (*n* = 27), who did not have a follow-up for more than 3 years (*n* = 32), and who did not undergo surgical treatment for various reasons (*n* = 5) were also excluded. Six patients who needed two or three debridement procedures before reimplantation were also excluded. Finally, 151 patients who consecutively received two-stage exchange total knee arthroplasty with reimplantation for chronic PJI by two senior surgeons and who had a minimal 3-year follow-up were enrolled and retrospectively reviewed (Fig. 1). Demographic data, radiographic data, serologic markers, synovial fluid analysis, and microbiological data were assessed in all patients.

### Treatment protocol

After chronic PJI diagnosis, surgical treatment first required implant removal and debridement of infected and necrotic tissue. Intraoperatively, a minimum of three tissue cultures were collected to assess presence of bacterial, fungal, and mycobacterial cultures. Antibiotic cement-articulated spacer insertion was performed in all patients by mixing 1 g of vancomycin per 40 g of cement and 2–4 g of total vancomycin, depending on the size of femur and tibia of each patient. Following cement spacer insertion, an orthopedic infectious disease specialist was consulted for targeted antibiotic therapy based on each patient’s culture results. Intravenous antibiotic therapy was started after tissue samples were obtained intraoperatively. The mean duration of intravenous antibiotics was 6 weeks, and the mean duration from cessation of antibiotics to reimplantation was 3 weeks. During recovery period after first stage surgery, weight-bearing ambulation was not allowed in all patients. After a mean 3-week antibiotic-free period, patients with persistent infection signs, such as discharging wound, increasing CRP, and local infection, underwent further debridement or other surgical procedures and then those patients were excluded from this study. Reimplantation was performed when the wound did not have any infection sign, CRP normalization, and the patient’s general condition was suitable. Cement space removal and thorough debridement was performed during the second surgery stage before reimplantation. Condylar constrained knee prostheses (LCCK revision, Zimmer, Warsaw, USA) and hybrid stem fixation technique methods were used in all cases of revisional total knee arthroplasty. Microbial culture study was routinely performed in all reimplantation. Intravenous (IV) antibiotics were administrated for 1 week after surgery, and oral antibiotics were prescribed for 2–4 weeks according to the identified organisms.

### Outcome measurements

The primary outcome was successful infection control of two-stage exchange revision TKA, such as (1) infection eradication and healed wound without drainage, fistula, recurrent effusion or pain; (2) no subsequent surgical intervention for infection (debridement, salvage procedures such as arthrodesis) after reimplantation; and (3) no PJI-related comorbidity such as sepsis or abscess. If successful infection control was not achieved within 3 years of reimplantation, it was defined as early septic failure. Of the 151 total enrolled patients, early failure was observed in 55 patients; and among them, 48 patients (87.2%) could be diagnosed as early septic failure. And 103 patients (68.2%) were in the successful treatment group. Confounding variables such as prior TKA history, medical history, radiologic septic loosening, presence of drainage sinus, and microorganism data were collected.

### Statistical analyses

The chi-squared test and *t*-test were performed to compare the successful treatment group and the early septic failure group. Multivariate logistic regression was performed to estimate independent risk factors with 95% confidence intervals (CI) for early septic failure. Statistical analyses were performed using SPSS software for Windows (Version 20.0, SPSS, Chicago, IL, USA), and *p*-values < 0.05 were considered significant.

## Results

A total of 151 patients with two-stage exchange revision TKA for PJI were analyzed and underwent an average duration of 5.3 years follow-up after reimplantation. Early septic failures occurred within 3 years of reimplantation in 48 patients (31.8%), and the mean time to early septic failure after reimplantation was 16 months (range: 1–35 months).

Demographics, baseline characteristics, and laboratory and microorganism data are presented in Table 1. The proportion of male patients (16/48, 33.3% versus 17/103, 16.5%; *p* = 0.02), fungal infection (7/48, 14.6% versus 5/103, 4.9%; *p* = 0.04), mycobacterial infection (5/48, 10.4% versus 1/103, 1%; *p* = 0.006), culture-positive at reimplantation (11/48, 22.9% versus 5/103, 4.9%; *p* = 0.001), and presence of a sinus tract in the external wound (15/48, 34.9% versus 11/103, 10.7%; *p* = 0.02) were significantly higher in the early septic failure group than the successful treatment group. No other differences in age, body-mass index (BMI), duration of primary TKA to PJI, erythrocyte sedimentation rate (ESR) and CRP at PJI diagnosis, synovial WBC and polymorphonucleocyte (PMN) at PJI diagnosis, or presence of radiologic septic loosening were found between the two groups (Table 1).

**Table 1:** Patient demographics and clinical characteristics

	Successful treatment group (*n* = 103)	Early septic failure group (*n* = 48)	*p* value
Age, mean ± standard deviation (SD) (years)	73.15 ± 5.5	71.1 ± 7.8	0.067
Male	17 (16.5%)	16 (33.3%)	0.02
BMI, mean ± SD (kg/m^2^)	25.52 ± 3.9	25.76 ± 3.8	0.729
Duration primary to revision, mean ± SD (month)	49.6 ± 58.3	37.0 ± 55.6	0.212
ESR, mean ± SD (mm/h)	73.6 ± 33.3	78.0 ± 33.0	0.451
CRP, mean ± SD (mg/L)	84.9 ± 102.1	59.8 ± 87.5	0.143
Synovial WBC, mean ± SD (cells/uL)	44771 ± 49167	47606 ± 79018	0.819
Synovial PMNs, mean ± SD (%)	83.9 ± 19.8	82.2 ± 21.2	0.700
Known disease			
Hypertension	79 (76.7%)	34 (70.8%)	0.439
Coronary heart disease	28 (27.2%)	10 (20.8%)	0.402
Diabetes mellitus	31 (30.1%)	16 (33.3%)	0.689
Stroke	10 (9.7%)	5 (10.4%)	0.892
Chronic kidney disease	18 (17.5%)	6 (12.5%)	0.436
Intraoperative organism			
Single positive culture	52 (50.5%)	23 (47.9%)	
Multiorganism positive culture	12 (11.7%)	11 (22.9%)	
Negative	39 (37.9%)	14 (29.2%)	
Fungal infection	5 (4.9%)	7 (14.6%)	0.04
Mycobacterial infection	1 (1%)	5 (10.4%)	0.006
Culture positive at reimplantation	5 (4.9%)	11 (22.9%)	0.001
Radiologic septic loosening	32 (37.2%)	20 (47.6%)	0.260
Sinus tract	11 (10.7%)	15 (34.9%)	0.002

Tables 2 and 3 show the intraoperative microbial culture results at first surgery (debridement, implant extraction, and cement spacer insertion) and at reimplantation surgery. Among patients in successful treatment group, 37.9% (39/103) were culture negative at the first surgery, and 11.7% (12/103) had polymicrobial infection wherein two or more microorganisms were detected. Methicillin-resistant coagulase-negative *Staphylococcus* species were isolated in 10 cases (9.7%), *S. aureus* in 20 cases (19.5%, including 8 cases with methicillin resistance), *Enterococcus* species in 9 cases (8.7%), and *Streptococcus* species in 7 cases (6.8%). Among patients with early septic failure, 29.2% (14/48) were culture negative at first surgery, and 22.9% (11/48) had polymicrobial infections. Methicillin-resistant coagulase-negative *Staphylococcus* species were isolated in 3 cases (6.3%), *S. aureus* in 11 cases (22.9%, including 6 cases with methicillin resistance), *Enterococcus* species in 1 case (2.1%), and *Streptococcus* species in 7 cases (4.2%). At the time of collecting the reimplantation intraoperative culture, only 5 cases (4.8%) were culture positive in the successful treatment group, whereas microorganisms were isolated in 11 cases (22.9%) in early septic failure group. Among these 16 cases, microorganisms different from those detected during previous surgery were found in four cases; they were all in the early septic failure group.

**Table 2:** Intraoperative microbial culture results from first surgery

	Successful treatment group (*n* = 103)	Early septic failure group (*n* = 48)	*P*
No growth	39 (37.9%)	14 (29.2%)	0.297
Methicillin-resistant coagulase-negative *Staphylococcus* species	10 (9.7%)	3 (6.3%)	0.480
Methicillin-resistant *Staphylococcus aureus*	8 (7.8%)	6 (12.5%)	0.350
Methicillin-sensitive *Staphylococcus aureus*	12 (11.7%)	5 (10.4%)	0.823
*Enterococcus* species	9 (8.7%)	1 (2.1%)	0.126
*Streptococcus* species	7 (6.8%)	2 (4.2%)	0.525
Other *Staphylococcus* species	1 (1%)	0 (0%)	0.493
*Corynebacterium* species	1 (1%)	1 (2.1%)	0.578
*Pseudomonas*	1 (1%)	0 (0%)	0.493
*Mycobacterium* species	1 (1%)	2 (4.2%)	0.190
Fungus	2 (1.9%)	3 (6.3%)	0.168
Polymicrobial organism	12 (11.7%)	11 (22.9%)	0.008

**Table 3:** Intraoperative microbial culture results at reimplantation

	Successful treatment group (*n* = 103)		Early septic failure group (*n* = 48)	
	Total number	Different microorganism as previous culture	Total number	Different microorganism as previous culture
No growth	98 (95.1%)		37 (77.1%)	
Methicillin-resistant coagulase-negative *Staphylococcus* species	2 (1.9%)	0	1 (2.1%)	1
Methicillin-resistant *Staphylococcus aureus*	0		2 (4.2%)	1
*Enterococcus* species	0		1 (2.1%)	0
Other *Staphylococcus* species	0		1 (2.1%)	0
*Mycobacterium* species	0		2 (4.2%)	0
Fungus	0		2 (4.2%)	0
Polymicrobial organism	3 (2.9%)	0	2 (4.2%)	2

Table 4 shows the variables associated with early septic failure after two-stage exchange revision TKA in multivariate analysis. After accounting for potentially confounding variables, we found that male patients [odds ratio (OR): 2.753, 95% CI 1.009–6.893, *p* = 0.031], fungus or mycobacterial infection (OR: 5.224, 95% CI 1.481–18.433, *p* = 0.01), and being culture positive at reimplantation (OR: 4.407, 95% CI 1.255–15.480, *p* = 0.021) were independently associated with early septic failure after two-stage exchange revision TKA (Table 4).

**Table 4:** Multivariate logistic regression of independent risk factors of early septic failure after two-stage exchange total knee arthroplasty in periprosthetic joint infection

Factor	Multivariate analysis	
	OR (95% CI)	*p* value
Age	0.952 (0.890–1.019)	0.154
Sex		
Female	1.00	
Male	2.753 (1.099–6.893)	0.031
BMI	1.023 (0.927–1.129)	0.651
Intraoperative culture result after first surgery		
No growth	1.00	
Single-culture positive	1.398 (0.356–5.489)	0.631
Polymicroorganism	1.237 (0.366–4.182)	0.732
Fungus or mycobacterial infection	5.224 (1.481–18.433)	0.010
Reimplantation culture positive	4.407 (1.255–15.480)	0.021
Radiologic septic loosening	0.628 (0.289–1.364)	0.240

## Discussion

Two-stage exchange revision total knee arthroplasty is considered the most successful surgical treatment among several options for chronic PJI; however, the high early septic failure rate of two-stage exchange revision TKA remains a problem, and surgical treatment for chronic PJI is still challenging [7, 25–27]. This study investigated the surgical outcomes of two-stage exchange revision TKA for chronic PJI and identified risk factors that affect early septic failure after two-stage exchange revision TKA for chronic PJI. The early septic failure rate within postoperative 3 years in this study was quite high at 31.8% (48/151) due to the common characteristics of tertiary hospitals. Treatment success rates after two-stage exchange revision TKA for chronic PJI were varied depending on the microorganism type from intraoperative microbial cultures. After controlling for relevant confounding variables, being culture-positive at reimplantation, fungus or mycobacterial infection, and male patients were independently associated with an increased risk of early septic failure after two-stage exchange revision TKA.

Being culture positive at reimplantation was an independent risk factor of early septic failure after two-stage exchange revision TKA, and it was about 4.4 times more likely to cause early septic failure after two-stage exchange revision TKA than being culture-negative at reimplantation, despite normal serum tests. The frequency of culture-positive status at reimplantation was 10.6% (16/151), which is close to the range reported from other studies (12–25%) [28–30]. Being culture positive at reimplantation, as described by Tan et al. [28], occurs because only the dominant microorganism was detected in polymicrobial infection, which is associated insufficient antibiotic treatment. Additionally, because being culture-positive at reimplantation may be associated with incomplete surgical debridement or emergence of new resistant microorganisms from long-term antibiotic treatment, these infections are more likely to cause to early septic failure than other types. Therefore, in this case, long-term suppressive antibiotic therapy and additional biofilm-active antibiotics could be necessary for achieving treatment success.

Fungal or mycobacterial infections were also found to be independent risk factors of early septic failure after revision TKA for chronic PJI. Fungal and mycobacterial infection are known as difficult-to-treat pathogens [31, 32], and many cases appear culture negative because it is difficult to detect pathogens in the first surgery [33]. Although the total number of fungal or mycobacterial infections in this study was small, the rate of early septic failure after two-stage revision TKA was also relatively high.

Despite the small number of patients in this study, the rate of early septic failure after two-stage revision TKA for chronic PJI was high in male patients. Several studies have shown that male patients have a higher rate of postoperative complications, including mortality, surgical site infection, and PJI, after TKA [34–36]. After accounting for potentially confounding variables, male patients were associated with early failure after two-stage revision TKA of chronic PJI. Lingde et al. explained that the reason for the high probability of PJI in male patients was that male patients may more active than female patients, so may potentially cycle their implant in greater numbers and induce a higher chance of infection [36]. Awareness of these findings could assist in risk stratification and help surgeons optimize patients’ preoperative risk when planning two-stage revision TKA for chronic PJI.

Radiologic septic loosening, including chronic inflammatory pathways, could be associated with biofilm-related infection and propagation into larger biofilm formation [37]. We did not find radiologic septic loosening to be an independent risk factor in this study. Furthermore, culture-negative PJI was not an independent risk factor for early septic failure after two-stage revision TKA for chronic PJI. Culture-negative PJI is difficult to treat because it is challenging to determine the appropriate antibiotics for the present microorganisms. However, in our study, presence or absence of microbial identification during reimplantation was more important for surgical outcomes of two-stage exchange revision TKA in chronic PJI.

Several factors, such as microorganism, antibiotics, and surgical and patient factors, could affect the overall surgical outcomes of two-stage revision TKA in chronic PJI. In this study, because these factors influenced the surgical outcomes in a complex way, they should all be considered during the whole treatment process. Additionally, by properly analyzing preoperative risk factors, additional surgical treatment such as three-stage surgery that includes explantation surgery and spacer change surgery before reimplantation or long-term antibiotic treatment should be considered for patients with a relatively high risk of early septic failure [31, 38]. And application of genetics, such as next generation sequencing, may be necessary for accurate microorganism detection.

This study has several limitations. First, it has a retrospective design and a relatively small patient sample because of the low frequency of patients with chronic PJI, which could be associated with an analysis bias. In addition, since the mean BMI of patients in this study was relatively low at 25.6, the effects of obesity, hypertension, diabetes mellitus, and other factors related to BMI in PJI may have been underestimated. Second, our study was performed at two urban tertiary referral hospitals and may therefore not be broadly generalizable. Third, antibiotic treatment varied across all patients, including empirical broad-spectrum antibiotics for culture-negative patients, different total antibiotic treatment periods, antibiotic holidays before second surgery, and oral antibiotic period after second surgery. This variance may have influenced the study results. Fourth, intraoperative frozen biopsy at reimplantation was performed only in some patients, so it was not included in the variable.

In conclusion, male patients, fungus or mycobacterial infection, and being culture-positive at reimplantation were independently associated with increased risk of early septic failure after two-stage exchange revision TKA despite normal CRP values prior to reimplantation. These results will help surgeon optimize their preoperative evaluations and decrease the early septic failure rate after two-stage revision TKA for chronic PJI. Further prospective and high-quality studies are needed to determine the risk factors of two-stage exchange revision TKA for chronic PJI.

## Data Availability

The datasets analyzed during the current study are not publicly available due to the high volume of data but are available from the corresponding author on reasonable request.
